# Downhole Applications of Magnetic Sensors

**DOI:** 10.3390/s17102384

**Published:** 2017-10-19

**Authors:** Chinthaka P. Gooneratne, Bodong Li, Timothy E. Moellendick

**Affiliations:** Drilling Technology Team, Exploration and Petroleum Engineering Center—Advanced Research Center (EXPEC-ARC), Dhahran 31311, Saudi Arabia; bodong.li.1@aramco.com (B.L.); timothy.moellendick@aramco.com (T.E.M.)

**Keywords:** fluxgate magnetometer, nuclear magnetic resonance, magnetic sensors, drilling technology, oil and gas, petroleum, downhole, harsh environment, high pressure high temperature (HPHT)

## Abstract

In this paper we present a review of the application of two types of magnetic sensors—fluxgate magnetometers and nuclear magnetic resonance (NMR) sensors—in the oil/gas industry. These magnetic sensors play a critical role in drilling wells safely, accurately and efficiently into a target reservoir zone by providing directional data of the well and acquiring information about the surrounding geological formations. Research into magnetic sensors for oil/gas drilling has not been explored by researchers to the same extent as other applications, such as biomedical, magnetic storage and automotive/aerospace applications. Therefore, this paper aims to serve as an opportunity for researchers to truly understand how magnetic sensors can be used in a downhole environment and to provide fertile ground for research and development in this area. A look ahead, discussing other magnetic sensor technologies that can potentially be used in the oil/gas industry is presented, and what is still needed in order deploy them in the field is also addressed.

## 1. Introduction

Magnetic sensors have been used in an extraordinary number of applications over the years, in the fields as diverse as automation, automotive, aerospace, biomedicine, computers, security, robotics, smart grids and textile technologies [1,2,3,4,5,6,7,8,9,10,11,12,13,14,15,16,17,18,19], and their utilization continues to increase at a rapid rate due to the advancements made in the area of nano-/microfabrication. In this paper, the application of magnetic sensors in the oil/gas industry, a relatively unexplored area of research compared with some of the aforementioned applications, is presented. 

Declining resources have forced oil/gas companies to drill deeper in different directions, and in more extreme and unknown environments. Therefore, it is important to monitor and analyze downhole environments in real-time when drilling a well in order to make timely decisions to optimize efficiency as well as prevent costly errors. One of the main ways of maximizing access to an oil/gas reservoir is to drill directional wells [20,21]. Directional drilling is the intentional deviation of a well from a vertical path at a predetermined trajectory, which allows access to reservoirs that cannot be reached efficiently with a vertical well drilled from the surface and maximizing reachability inside a reservoir. Therefore, directional drilling is used to optimize the production of hydrocarbons. Moreover, by drilling multiple directional wells from a drilling platform rather than drilling several vertical wells the drilling cost, impact on the environment and health and safety issues can be reduced. When planning directional wells, there are many considerations that have to be taken into account, such as target location, shape and size, well trajectory, geological formations, adjacent wells and rig surface facilities. The deviation of the well has to be accurately controlled in order to keep the trajectory of the well within the prescribed angle in order to reach the intended target. Failure to accurately drill a directional well can result in a ‘dry hole’, and significant financial losses for the company, as well as impacting their business strategy. 

The oil/gas industry exploits the affordable, rugged, compact and reliable features of magnetic sensors, using them in harsh downhole environments. Fluxgate magnetometers (FGMs) and nuclear magnetic resonance (NMR) sensors play a significant role in optimizing well placement and completion resulting in maximum access to oil/gas reservoirs and higher production rates. In this paper we describe how FGMs and NMR sensors are utilized to obtain measurements inside wells during the drilling process so that wells can safely and efficiently reach oil/gas reservoirs located thousands of feet below the ground whilst also obtaining maximum access to these reservoirs. FGMs give the driller at the surface a means of navigating a well, whereas NMR sensors provide information about the geological characteristics of the formations being drilled through, in real-time. 

## 2. Downhole Magnetometers

### 2.1. Principles of Fluxgate Magnetometers

Since their inception in the 1930s, FGMs have been used to measure magnetic fields in a wide range of applications [22,23,24] and have recently progressed to solid-state sensors with the advancements made in micro/nanofabrication technology. Thorough reviews of FGMs can be found in [1,2,25,26,27]. Typical parameters of FGMs are shown in Table 1. Referring to Table 1, the magnetic field range, noise level and linearity allows FGMs to measure the Earth’s magnetic field, between 25 and 65 μT, and the sensitivity allows a measurable output with a 5 V power supply and simple signal processing. Moreover, the low temperature coefficient means that FGMs can be used when drilling wells with depths up to 20,000 feet, where temperatures can be as high as 230 °C. Several papers have demonstrated the stability of FGMs in a 180–250 °C temperature range [28,29]. Not only do FGMs have excellent noise characteristics compared with other magnetic sensors but they can be constructed easily according to well established design principles at low cost.

**Figure 1:**
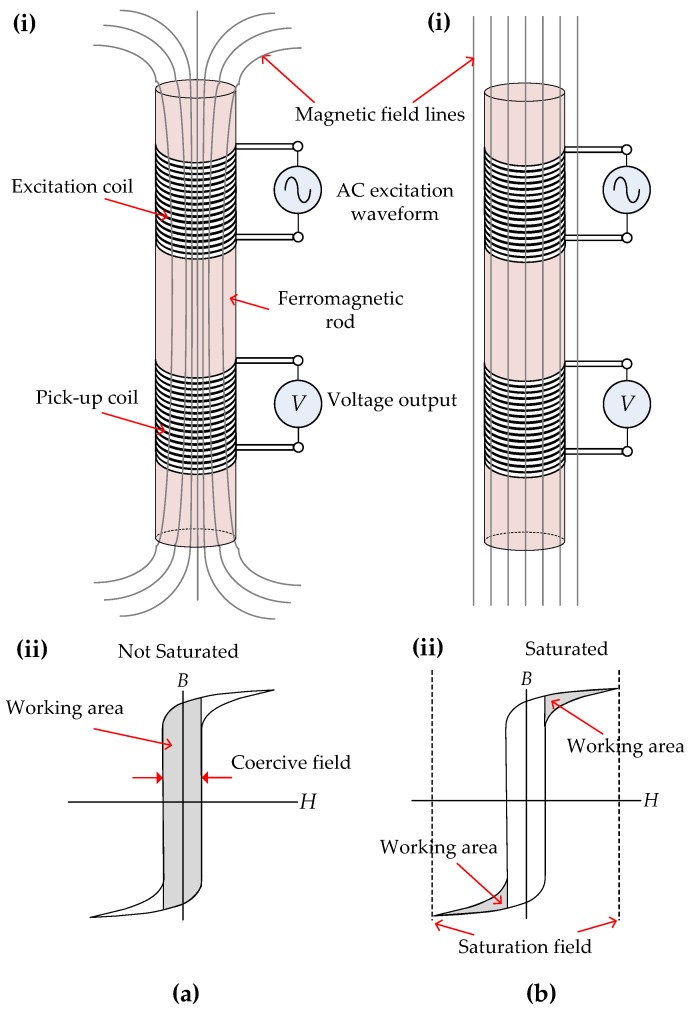
(**a**) Principle operation of a fluxgate magnetometer (FGM). (**i**) Application of an AC current and the induction of a magnetic flux density (*B*) in the rod and (**ii**) the corresponding *B*-*H* curve showing the grey area the FGM operates in during this excitation phase; (**b**) (**i**) The FGM in saturation mode where *B* has saturated and magnetic field (*H*) lines do not converge to the rod resulting in (**ii**) the FGM operating in the saturated area of the *B*-*H* curve.

The working principle of an FGM in its simplest form can be explained with reference to Figure 1. An FGM consists of two coils, an excitation and a pick-up coil, wound around a ferromagnetic rod as shown in Figure 1(ai,bi). The ferromagnetic rod is driven to saturation when a large alternating current (AC) is applied to the excitation coil by a waveform generator and a magnetic flux density (*B*) is induced in the rod, as shown in Figure 1(ai). As the rod is driven into saturation, as shown in Figure 1(bi), it becomes progressively more difficult for magnetic field (*H*) lines to pass through the rod and induce a *B*. This reluctance of the rod is sensed by the pick-up coil, which creates changes in the voltage of the pick-up coil. Since the rod is driven to saturation twice during each excitation cycle, the second harmonic of the output voltage of the pick-up coil is extracted by phase demodulation circuitry. When the FGM is in the presence of an external *H* (*H_ext_*), such as the Earth’s magnetic field, the induced *B* is distorted. This distortion is sensed by the pick-up coil causing a change in the output voltage; the magnitude corresponds to the strength of *H_ext_* and the phase to the orientation of the *H_ext_*. The magnetic hysteresis (*B*-*H*) curve in Figure 1(aii) shows the operation of the FGM in the linear region during excitation, and the *B*-*H* curve in Figure 1(bii) shows the operation of the FGM in saturation. The sensitivity of the FGM depends on the *B*-*H* curve, where a steeper magnetizing curve relates to a more sensitive FGM. The power consumption of an FGM depends on the coercivity and saturation fields as shown in Figure 1(aii,bii). Lower saturation coercivity fields mean lower magnetic fields, and hence lower excitation currents and power, required to drive the rod to saturation and back to zero after being saturated. The frequency response of an FGM depends on the time lag between the application of the excitation field and the response of the ferromagnetic rod. 

**Figure 2:**
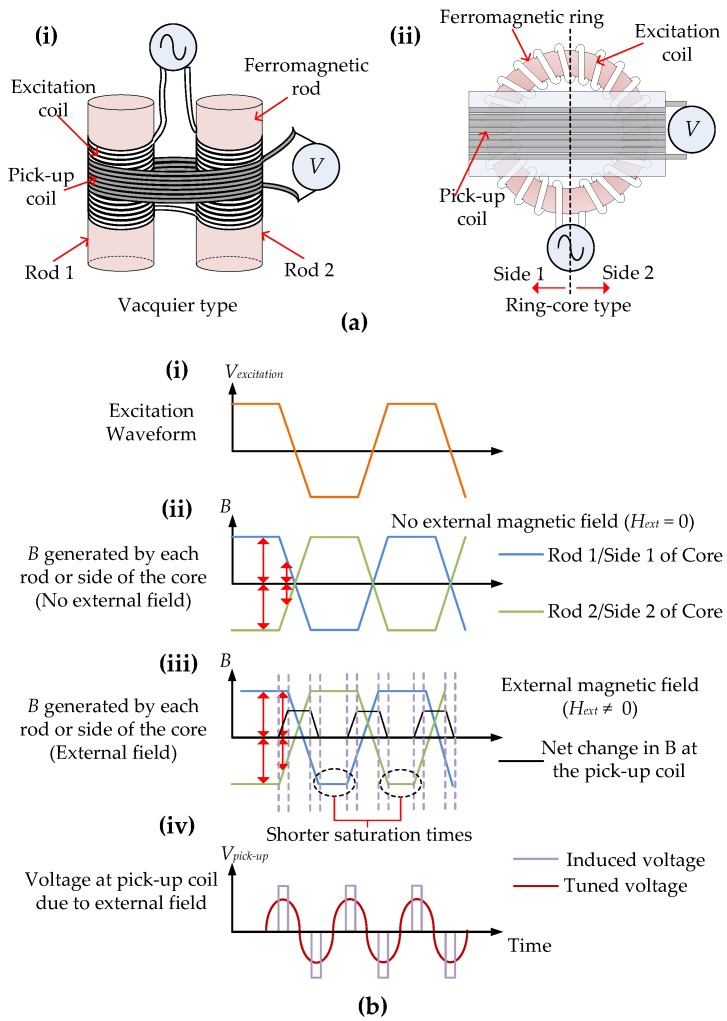
(**a**)Variants of an FGM. (**i**) Vacquier-type FGM with wires wound on both rods 1 and 2 in opposite directions to each other; (**ii**) Ring-core type FGM where the windings on side 1 of the core is in opposite direction to side 2; (**b**) (**i**) Application of an excitation waveform (orange waveform) to (**a**) (**i**) or (**a**) (**ii**); (**ii**) In the absence of an external magnetic field (*H_ext_*), *B* induced in rod 1/side 1 (blue waveform) is opposite in polarity to B induced in rod 2/side 2 (green waveform), so net magnetization and voltage (V) induced at the pick-up coil is zero; (**iii**) In the presence of an *H_ext_* the rod/core generating a magnetic field in the same direction as *H_ext_* have a shorter saturation time and there is a net change in B in the pick-up coil (black waveform); (**iv**) This net change in B induces a voltage in the pick-up coil (purple waveform). A clear amplified waveform (red waveform) can be obtained by tuning the pick-up coil [33].

In reality, for a single ferromagnetic rod, the pick-up coil will sense both the excitation voltage as well as the output voltage. This makes it challenging to filter out the second harmonic, obtain its phase and rectify it to obtain voltage proportional to the magnitude of the external field. In order to overcome this challenge two variants of the FGM, a Vacquier-type FGM, shown in Figure 2(ai), and a ring-core FGM, shown in Figure 2(aii), are commonly used [26,30,31,32]. Taking Figure 2a into account, the wires are wound on both rods in opposite directions to each other in a Vacquier-type FGM and, similarly for a ring-core FGM, the windings are such that on one half of the core they are in the opposite direction to the other half. When an excitation current is applied, the induced *B* in one rod or half of the core will have the opposite polarity to *B* in the second rod or the other half of the core. This results in a net magnetization of zero and an output voltage of zero at the pick-up coil. For example, Figure 2(bi,bii) show that when an excitation current (orange waveform) is applied, *B* increases with the current and reaches saturation at the peak of the excitation current. The *B* produced from both rods and both sides of the cores are mirror images of each other along the *x*-axis (blue and green waveforms) resulting in a net *B* of zero [33]. The voltages from both rods and sides of the core at the pick-up coil are also mirror images along the *x*-axis, are proportional to the rate of change of *B*, and increase and then reach zero at saturation as the rate of change of *B* is zero at saturation. However, when there is an *H_ext_*, the rod or the half-core that is generating an *H* in the same direction as *H_ext_* takes longer to come out of saturation, therefore the rod or the half-core generating an *H* in the opposite direction comes out of saturation sooner. This can be seen in Figure 2(biii) from the short and long saturation times for each rod or half-core every half cycle of the waveform. This creates a net change in *B* in the pick-up coil (black waveform), which induces a voltage in the pick-up coil (purple waveform), as shown in Figure 2(biv). A clear amplified waveform (red waveform) can be obtained by tuning the pick-up coil. 

More recently, miniature FGMs have been fabricated using complementary metal-oxide-semiconductor (CMOS), micro-fabrication and printed circuit board PCB methods [34,35,36,37]. Their size, compactness, low power consumption and the possibility of integration with electronics into integrated circuit (IC) chips make them ideal candidates for portable devices. However, one of the major drawbacks of miniature FGMs is the limited number of turns possible in the excitation and pick-up coils during the fabrication process. The limited number of turns in the excitation coil in a miniature FGM results in the rod or core not being properly saturated, and in a pick-up coil leads to lower sensitivities than traditional FGMs. Higher amplitudes and frequencies of the excitation current can be used to compensate for this drawback but at the cost of higher power consumption. Moreover, compared to traditional simply-wound FGMs there is a higher cost associated with microfabrication of miniature FGMs. 

### 2.2. Navigating a Well Using Magnetometers 

**Figure 3:**
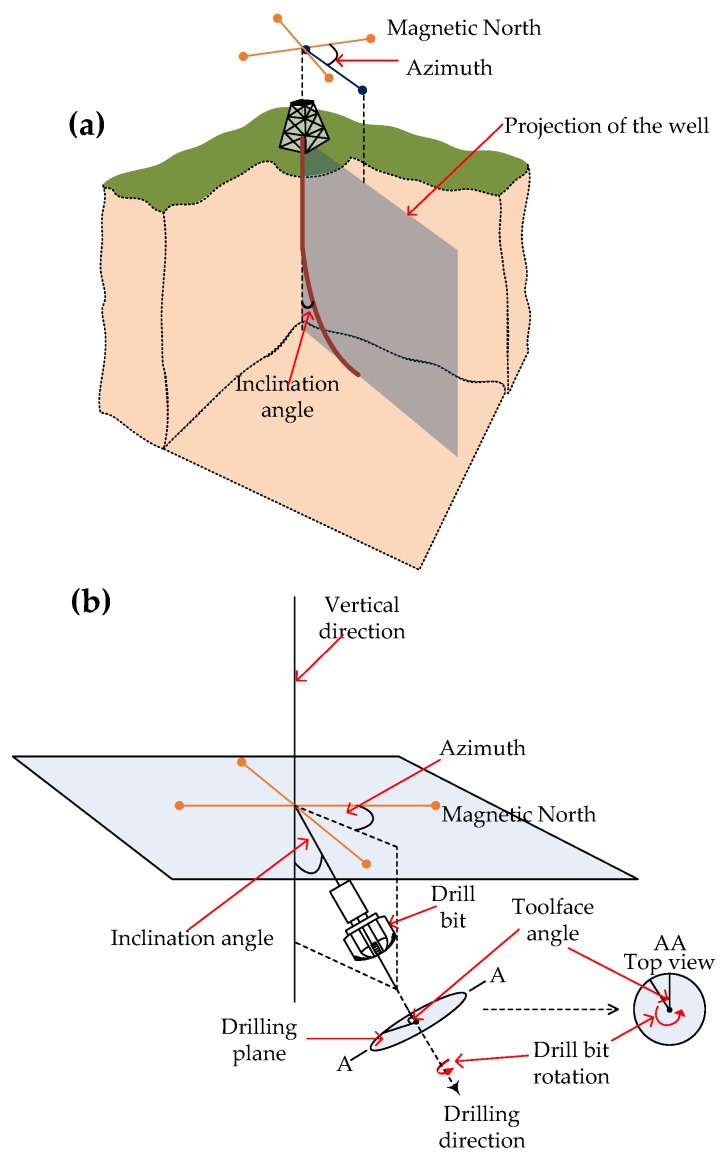
(**a**) Azimuth and inclination when drilling a directional well; (**b**) The azimuth of a directional well is the deviation from the magnetic north and the inclination is the deviation from the vertical direction of the well. The toolface is the angle the drill bit rotates on the drilling plane from an initial reference point.

In directional drilling, the well is deviated from a vertical trajectory to a trajectory that is kept within prescribed limits of azimuth and inclination to reach a final landing point as shown in Figure 3a. Directional drilling is performed so that the final landing point, typically a reservoir, can be reached when it is below a populated area or areas inaccessible due to obstructions such as mountains or rivers. Directional drilling allows multiple wells to be drilled from a single vertical well and significantly increases the access and exposure to a reservoir compared with vertical drilling. As Figure 3b shows, directional drilling is a three dimensional process where the azimuth is the deviation from the magnetic north in the horizontal plane, and the inclination of the well is the angle the well deviates from the vertical direction, represented as zero degrees. The azimuth is defined as the orientation of the well, measured clockwise with respect to the magnetic north. The line along the vertical direction is always parallel to the Earth’s gravitational field. The toolface, as shown in Figure 3b, is the angle the drill bit rotates on the drilling plane from an initial reference point.

The earliest directional drilling tools, such as lowering an acid bottle into a well to etch an acid ring on the bottle and the Totco mechanical drift recorder, only measured the inclination of a well [38]. Magnetic single and multi-shot surveys were the first instruments to measure both inclination and azimuth, and consisted of a magnetic compass, inclinometer and a camera controlled by an electronic timer [39]. These single and multi-shot devices had to be run on wireline down a well or dropped down the drillstring assembly and retrieved after pulling the drillstring out of the well. 

**Figure 4:**
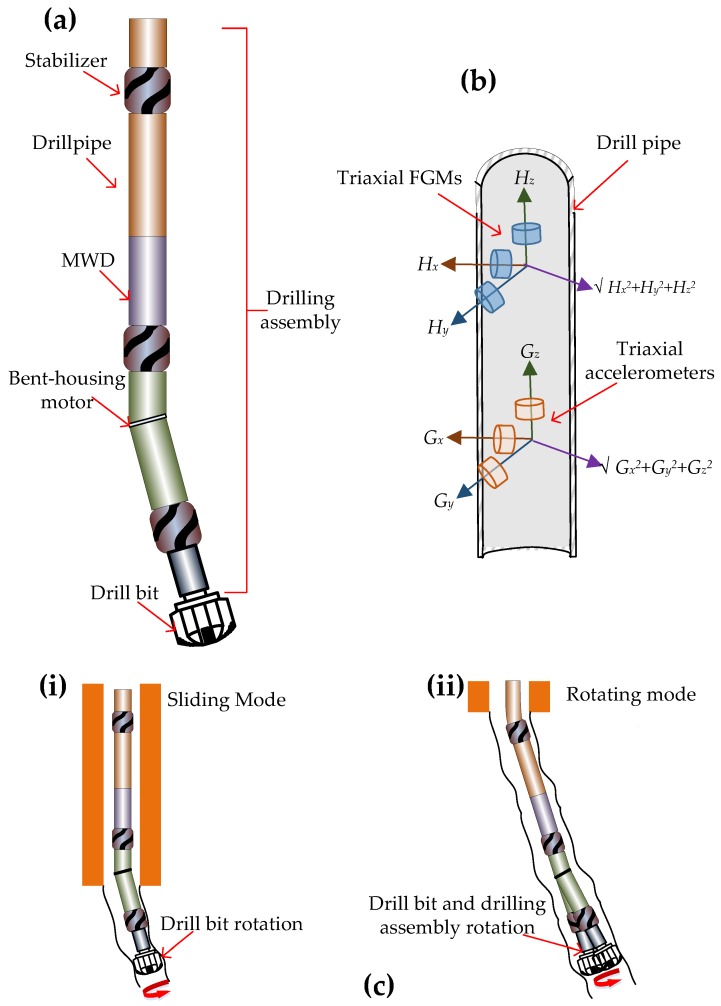
(**a**) Drilling assembly for directional drilling with an MWD unit consisting of FGMs and accelerometers, as shown in (**b**), to obtain azimuth and inclination measurements of the well, a bent-housing motor, as shown in (**c**) (**i**), that initiates the trajectory of the well being drilled, and stabilizers that allow side force to be generated at the bit. Once the desired trajectory is obtained the whole drilling assembly and the bent-motor drills ahead as shown in (**ii**).

Early well deviating methods included setting whipstocks, jetting tools and build, drop and pendulum BHA assemblies [38,40,41,42,43,44]. However, the advent of the downhole mud motor and the rapid development of compact, rugged sensors along with the mud pulse telemetry method of transmitting data from downhole to the surface allowed the azimuth and inclination to be measured in real-time. The most established method of using this measurement while drilling (MWD) technique is using the configuration shown in Figure 4a, which has a bent-housing motor, several stabilizers and a MWD unit. The bent-housing motor has a hydraulic motor that is driven by the drilling fluid flowing through the drilling assembly. 

The MWD unit includes tri-axial FGMsand tri-axial accelerometers, as shown in Figure 4b and the mud-pulse telemetry system (not shown), which is located above the fluxgate magnetometers and accelerometers. The stabilizers are used to control contact with the wellbore and form a fulcrum with the hydraulic motor behind it acting as a lever, thus allowing side force to be generated at the bit. The bend is adjusted according to the angle of the well being drilled and is normally set anywhere between 0° and 2° but sometimes as high as 3°. Initially only the hydraulic motor powers the drill bit and there is no rotation of the drilling assembly above the drill string, as shown in Figure 4(ci). The motor can be oriented in any desired manner to build angle, drop angle or turn. Once the desired trajectory of the well is attained the entire drilling assembly and the bit are rotated to drill straight ahead as shown in Figure 4(cii). 

The Earth’s magnetic field has a different strength and orientation at every location on Earth and this field is measured using tri-axial FGMs while the inclination of a well is obtained by measuring the gravitational field by tri-axial accelerometers. FGMs are used to measure the toolface when the well is vertical (0° inclination) as the gravitational field will be constant, and accelerometers are used to measure the toolface when the well is horizontal (90° inclination). Any toolface measurement between an inclination of 0° and 90° is performed by both FGMs and accelerometers. Generally the directional MWD crosses over from magnetic tooolface to gravitational toolface at angles from 3° to 5°. The position *P* of the drill bit in a well being drilled can be obtained at any time in terms of the magnetic field, inclination and toolface as shown below [45]:

(1) $$P=\arctan\;\left(\frac{-\left(H_{x}\sin\varphi +H_{y}\cos\varphi \right)}{H_{z}\sin\theta +\cos\theta \left(H_{x}\cos\varphi -H_{y}\sin\varphi \right)}\right)$$

and:

(2) $$\theta =\arctan\;\left(\sqrt{\frac{G_{x}{}^{2}+G_{y}{}^{2}}{G_{z}{}^{2}}}\right)$$

and:

(3) $$\varphi =\arctan\;\left(-\frac{G_{y}}{G_{x}}\right)$$

where *θ* is the inclination, *ϕ* is the toolface angle and *G_x_*, *G_y_* and *G_z_* are the orthogonal gravitational vectors measured by the accelerometer.

While drilling, there are predetermined survey points along the well where information about the azimuth, inclination and toolface is obtained. Values at a given survey station are combined with previous values to obtain the well trajectory, where the computations are based on mathematical assumptions. This data is transmitted to the surface so that the driller on the surface knows the exact direction in which the well is being drilled. 

**Figure 5:**
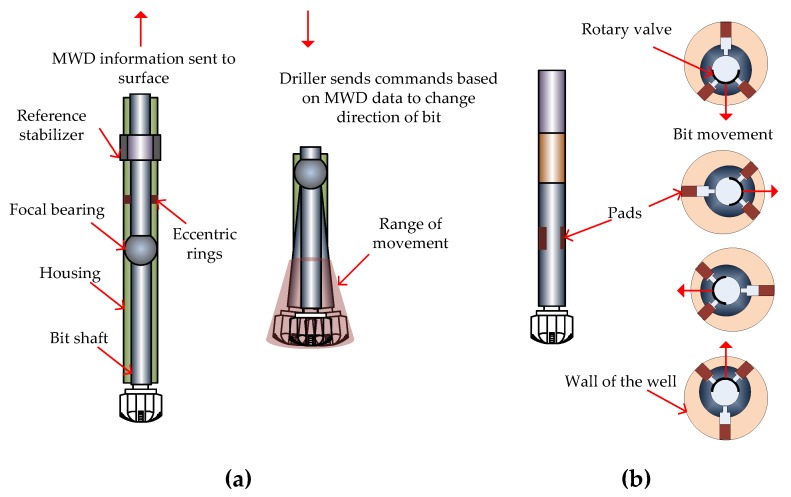
(**a**) Point-the-bit system, where the bit shaft is bent relative to the rest of the drilling assembly. The bend orientation is controlled by a servomotor that rotates at the same speed as the drilling assembly but in the opposite direction so that the toolface orientation is non-rotating; (**b**) Push-the-bit system, where the pads are actuated by a flow and pushed out against the wall of the well being drilled to direct the drillstring assembly in a desired direction.

The sliding and rotating method, while established, is time consuming since the rate of penetration into the earth is significantly lower during the sliding mode compared to the rotating mode. The rotary steerable system (RSS) is a technology, introduced approximately 20 years ago, that allows the constant rotation of the entire drilling assembly while drilling a directional well, therefore increasing the average rate of penetration [46,47,48]. An increase in the rate of penetration means less time to drill a well and, hence, significant savings for the company. RSS is more expensive than sliding/rotating methods and therefore, how it can be optimized for a given well to increase the rate of penetration has to be carefully planned. Production potential of the well, earth formations, depth of a well, drill bit compatibility and expected hole problems are some of the factors that must be considered. There are two main RSS technologies, point-the-bit, as shown in Figure 5a, and push-the-bit, as shown in Figure 5b. In point-the-bit systems the bit shaft is bent relative to the rest of the drilling assembly. The amount of bend depends on the commands the driller on the surface sends downhole, where the commands are based on the directional measurements, azimuth, inclination and toolface, sent by the MWD from downhole to the driller at the surface. A servomotor controls the bend orientation and the servomotor rotates at the same speed as the drilling assembly but in the opposite direction, thereby allowing the toolface orientation to stay non-rotating while the drilling assembly rotates. The reference stabilizer acts as a reference to create the deflection within the shaft and the trajectory of the well changes in the direction of the bend. In push-the-bit systems the pads, shown in Figure 5b, are actuated by the drilling fluid flow and pushed out against the wall of the well being drilled to direct the drillstring assembly in a desired direction. A rotary valve opens and closes the drilling fluid supply to the pads and the timing and magnitude of pad actuation depends on the commands the driller sends downhole from the surface based on the directional data sent to the surface by the MWD. 

One of the drawbacks of FGMs is that they have to be run within a nonmagnetic environment since the measured azimuth is with reference to the magnetic north. Therefore, any magnetic interference due to fields other than the Earth’s magnetic field will cause significant deterioration of magnetic azimuth accuracy. To overcome this issue FGMs are enclosed in a nonmagnetic drill cover and run inside a well. Depending on the ‘proposed azimuth’ and inclination, increasing amounts of non-magnetic drill collar are required to effectively isolate the FGMs. Vertical is the best case; 90° or horizontal due east or west is the most challenging. 

### 2.3. Field Results

**Figure 6:**
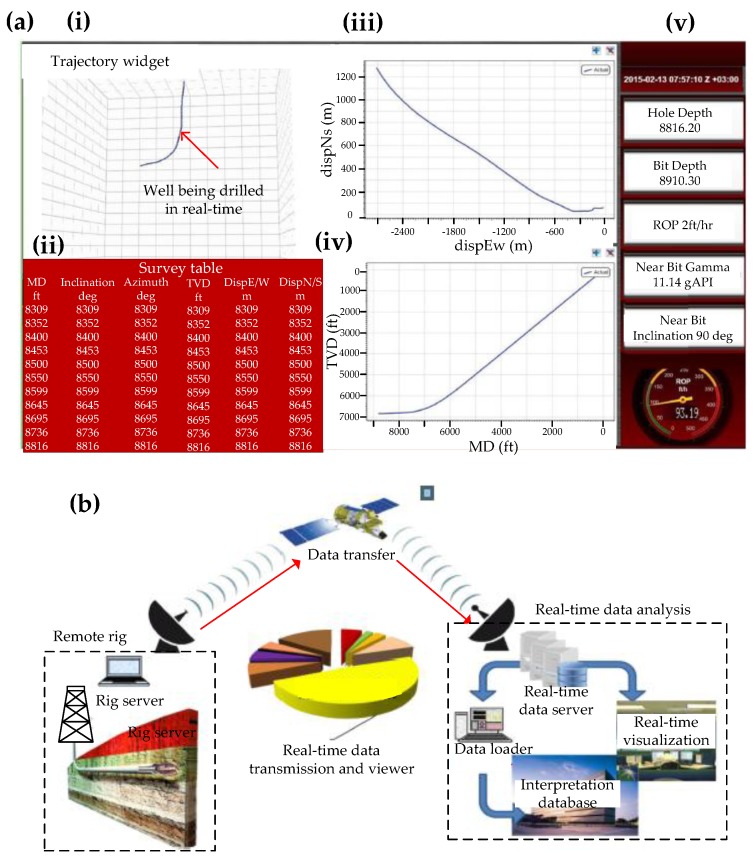
(**a**) Real-time directional drilling display available to a drilling engineer. (**i**) Real-time trajectory of a well in 3D where (**ii**) shows the real-time azimuth, inclination and measured depth (MD) and total vertical depth (TVD) of the well as well as the east-west and north-south coordinates (polar coordinates); (**iii**) Graph showing the displacement of the well in polar coordinates, where they can be used to calculate distance (departure) from the surface and direction (azimuth); (**iv**) Graph showing the measured depth, which is the total length of the well, and the true vertical depth, which is the vertical distance from the surface to the final depth of the directional well. A change from a vertical to a directional trajectory can be seen after an MD of 6500 m; (**v**) Real-time drilling parameters showing the well depth, current depth of the bit, the rate of penetration of the bit into the formation, gamma ray readings to indicate the formation the well is being drilled through and the inclination of the well; (**b**) Navigation workflow that shows data transfer from a remote rig server to a real-time data server at the analysis center. The real-time data server then sends this data to a real-time visualization and interpretation center so that the drilling engineers can be guided during drilling the directional well to accurately reach the final target into a reservoir [49].

Figure 6 shows real-time data transmission and visualization through a drilling assembly navigation software [49]. There is a real-time communication system, ‘Rig Chat’, linking the navigation experts in the office, rig personnel, and service companies, enabling them to communicate with each other to monitor and guide navigation operations in an efficient manner. As Figure 6(ai) illustrates, there is a real-time directional drilling display available to a drilling engineer to show the actual trajectory of the well in 3D. Figure 6(aii) shows the the real-time azimuth, inclination, measured depth (MD) and total vertical depth (TVD) of the well along with the east-west and north-south coordinates (polar coordinates). MD is the total length of the well and TVD is the vertical distance from the surface to the final depth of the directional well. The east-west and north-south polar coordinates shown in Figure 6(aiii) calculate distance (departure) from the surface and direction (azimuth). Figure 6(aiv) shows the TVD vs. MD graph, where a change from a vertical to a directional trajectory can be seen after 6500 m. Figure 6av shows real-time drilling parameters such as the well depth, current depth of the bit, the rate of penetration of the bit into the formation, gamma ray readings to indicate the formation the well is being drilled through and the inclination of the well. The navigation data workflow shown in Figure 6b not only involves monitoring the well in real-time but also correlating and interpreting the data obtained by the downhole sensors, which measure drilling dynamics such as ROP, torque and downhole environmental data such as pressure, temperature and density. The workflow involves transferring data from a rig server in a remote rig to a real-time data server at the analysis center. The real-time data server then sends this data to a real-time visualization and interpretation center so that the drilling engineers on the remote rig can be guided remotely to accurately reach the final target into a reservoir. 

**Figure 7:**
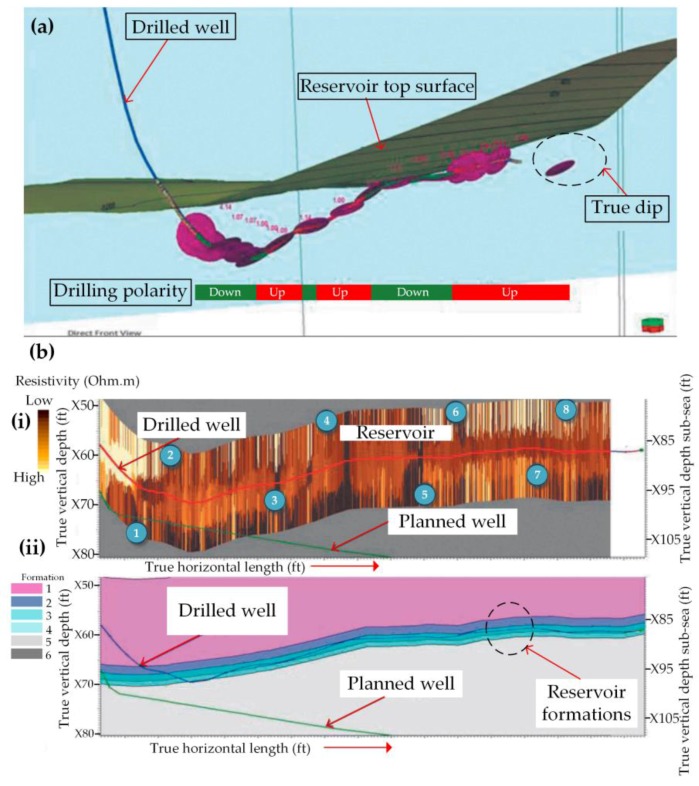
(**a**) Real time 3D visualization of directional drilling in a very thin but high quality reservoir, where the ‘dip’ refers to the orientation of the reservoir bedding. The drilling polarity plots indicate whether the well was being drilled stratigraphically up or down; (**b**) (**i**) Formation resistivity image log showing the well being steered inside the reservoir. (1) Well being drilled at an inclination of 89°; (2) Inclination increased to 91° based on resistivity and mobility logs; (3) Inclination kept at 90.5° as formation apparent dip was estimated from the real-time resistivity log to be 0.4° dipping towards the surface; (4) Resistivity image and other logs indicated that the high mobility was below the bit so an inclination of 90° was held to gradually drill downward into the target; (5) Inclination was dropped to 89.5° based on resistivity logs; (6) Inclination was built and held at 90° and the formation dip was also estimated to be almost flat; (7) Dip was estimated to be dipping away from the surface at about +0.5° so inclination was dropped to 89.5°; (8) Since the reservoir dip was estimated to be flat the well was drilled with an inclination of 90° until final target was reached; (**ii**) Plot corresponding to (**i**) showing the formations drilled through, where formation 1 is the top, and the actual well drilled with the aid of logging vs the planned well trajectory before drilling [50].

Figure 7a shows a 3D visualization of real-time dip data, with drilling polarity indicated on the trajectory when steering along a thin reservoir interval with high permeability [50]. The dip and inclinometer data were not only used to steer the well and update the real-time trajectory but were also used to calculate the true stratigraphic index of the well and its relative angle with respect to the local structural dip to accurately profile logging data. The trajectory shows several dips through which the well was steered, below the top surface of the reservoir. The drilling polarity plots indicate if the well is being drilled stratigraphically up or down and are useful when steering wells in complex reservoirs or simple but very thin interval reservoirs such as in this example. 

Figure 7(bi) shows the well being steered inside the thin reservoir with the aid of resistivity logs obtained while drilling. In (1) the trajectory of the well has an inclination of 89° and in (2) it was increased to 91° based on the resisitivity logs and mobility information. In (3) resistivity logs indicated that the reservoir formation apparent dip was 0.4°, dipping towards the surface, so the inclination was kept to 90.5°. Increased mobility below the bit was indicated by resistivity and other logs so an inclination of 90° was held to gradually drill downward into the target. The drilling assembly was then pulled out of the well due to problems in the well and run in the well again. In (5) inclination was dropped to 89.5° and then once the resistivity logs confirmed that the bit was in the target reservoir again the inclination was built and held at 90° since the formation dip was estimated to be almost flat as shown in (6). After drilling ahead the dip was estimated to be dipping away from the surface at about +0.5° so the inclination was dropped to 89.5°. Further drilling showed that the reservoir dip was flat so the rest of the well was drilled at an inclination of 90° until the target depth was reached. Figure 7(bii) shows the different reservoir formation layers drilled through, where formation 1 is the top, and the actual well drilled with the aid of logging vs the planned well trajectory before drilling. There is significant deviation from the originally planned well but real-time logging data correlated to the position of the well aided navigation of the well through the reservoir, maximizing the reach into the reservoir. 

**Figure 8:**
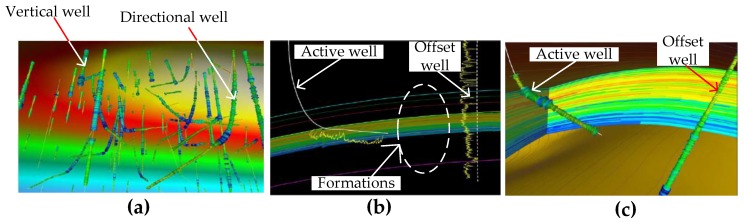
(**a**) 3D visualization of drilled wells in a field. Drilling in such a complex field with many vertical and directional wells requires careful planning to avoid well collisions; (**b**) Cross-sectional view of an active well being drilled in the vicinity of an offset well; (**c**) 3D view of the same well in (**b**) showing the active well approaching the offset well [51].

Figure 8a shows the complex setting of directional wells in a field [51]. When drilling new directional wells to reach reservoirs, avoiding collision with wells already drilled in the field is imperative. Figure 8b shows the cross-section of an active drilling well and an offset well located nearby, and Figure 8c shows a 3D visualization of Figure 8b. The position of the active well given by the directional sensors aids the driller to clearly plan the trajectory of the well taking into account other wells in the vicinity. These previously drilled wells, called offset wells, can be vertical or directional and their locations are built into the navigation model. By knowing their location the driller can adjust the trajectory of the well so that the active well is drilled at a safe distance from the offset wells to avoid collisions so as not to affect their production potential. Furthermore, while the active well is drilled at a safe distance to offset wells it is also important to ensure that the active well is navigated towards the reservoir in a trajectory that can optimize access to the reservoir. 

The errors due to combined/collective sensor error/accuracy can be determined by ellipses of uncertainty. An ellipsoid of uncertainty (EOU) error model, applicable to a basic MWD, was developed by the industry steering committee on wellbore survey (ISCWSA) and is the recognized and current industry standard for calculating well position uncertainty [52]. The EOU error model takes into account tools from different service companies and makes use of directional drilling software developed to integrate with subsurface applications to visualize well position uncertainty. 

**Figure 9:**
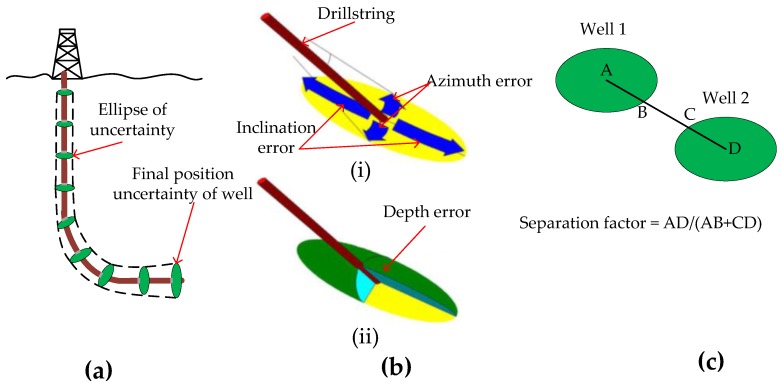
(**a**) The ellipses of uncertainty gets larger as the depth from the surface increases since errors from different sources are statistically independent, are cumulative and errors propagate in proportion to how far you are from the origin; (**b**) The lateral dimension of the ellipse of uncertainty is proportional to the azimuth error and the high side dimension is proportional to the inclination error; (**c**) Calculation of separation factor between two wells to avoid well collision [52].

The errors are calculated at well survey stations, placed a maximum of 100 ft apart, and are modeled as vectors based on three elements; the azimuth, inclination and the depth of the well. Toolface angle is also taken into account when modeling of the propagation of errors. Errors from different sources are statistically independent, are cumulative and propagate in proportion to how far you are from the origin. Therefore, the final position of the well can be anywhere within the final position uncertainty shown in Figure 9a. An error source is a physical phenomenon that influences the measurement obtained by a survey tool such as an MWD and is described by an error term [53]. An error model is therefore a set of error terms chosen to account for all the different error sources that affects a survey tool. The error terms in the EOU model are sensor errors, errors due to steel in the drilling assembly near the MWD, directional sensor assembly misalignments and magnetic dip and field strength uncertainties. 

As shown in Figure 9(bi), the lateral dimension of an ellipse of uncertainty is proportional to the azimuth error and the high side dimension is proportional to the inclination error. A thinner ellipse represents the case where the azimuth is more accurate than the inclination and a more spread-out ellipse represents the opposite case where the inclination is more accurate than the azimuth, where the latter example is more typical in the field. Figure 9(bii) shows the third component of the error, the measured depth of the well, resulting in an almond-like shape, elliptical in all three orthogonal planes.

EOUs are used to estimate the accuracy of hitting the target reservoir, especially when the reservoir is thin, and also more importantly to avoid collision of wells. Figure 1c shows the simplest method of evaluating the collision risk, a separation factor that is calculated in a plane at right angles to the well that has already been drilled. A separation factor of 1 indicates that the wells are just touching but doesn’t necessarily mean there would be a collision as a probability with 2 or 3 standard deviations are used when determining the size of the ellipses. However, the risk of collision increases as the overlapping region increases. Other methods of calculating the separation factor can be found in [52].

## 3. Nuclear Magnetic Resonance (NMR)

### 3.1. Principles of NMR

**Figure 10:**
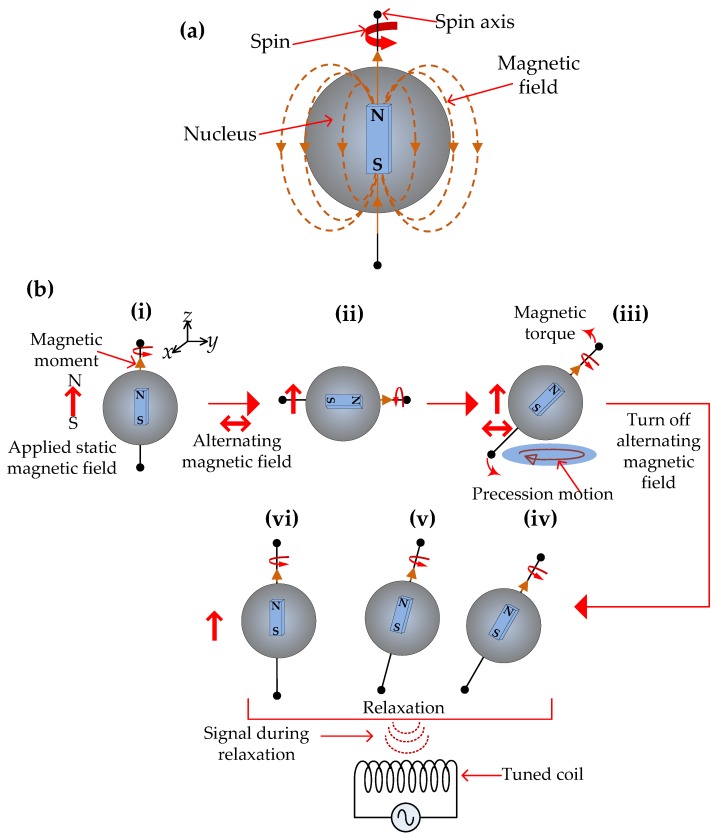
(**a**) A spinning nucleus emanating a magnetic field; (**b**) (**i**) Application of a static external magnetic field results in the orientation of the nucleus to be in the same direction as the external field. (**ii**) Application of an RF magnetic field perpendicular to the static field tips the nucleus away from the static field. (**iii**) When the nucleus is tipped away from the static magnetic field the orientation of its rotational axis changes leads to precession. (**iv**–**vi**) During the precession motion the nucleus emits RF waves that can be detected by a tuned coil and when the RF field is turned off the nucleus relaxes back to thermal equilibrium and aligns itself with the static magnetic field.

An atom nucleus can be thought of as a small bar magnet with a magnetic moment and a magnetic field since it has a positive charge and behaves as though it is spinning along a spin rotation axis, as shown in Figure 10a. In the absence of a strong external magnetic field the nucleus will be oriented in a random direction, even though it will be weakly aligned with the Earth’s magnetic field. When the nucleus is exposed to a large external static magnetic field the orientation of the nucleus will no longer be random and will be aligned with the direction of the external magnetic field, as shown in Figure 10(bi). The orientation will now only flip infrequently between the low and high energy states compared to when there was no static magnetic field. 

By applying a radio frequency (RF) magnetic field perpendicular to the static magnetic field the nucleus can be tipped away from the static magnetic field direction as shown in Figure 10(bii). The RF magnetic field can be applied as a series of short, pulses, usually in the range of microseconds. The irradiation energy in the RF magnetic field is equal to the difference in energies of the two nuclear spins with different orientations (low and high energy) and the absorption of this energy by the nuclear spins causes it to flip from higher to lower energy and lower to higher energy in a process known as resonance. The energy required for resonance depends on the strength of the static magnetic field *B*_0_, and can be obtained by the following equation [54]:

(4) $$\Delta E=\frac{\gamma hB_{0}}{2\pi }$$

where *h* is Planck’s constant (6.63 × 10^−27^ erg sec). The Bohr condition (ΔE=hv) enables the frequency *v*_0_ of the nuclear transition to be written as:

(5) $$v_{0}=\frac{\gamma B_{0}}{2\pi }$$

Equation (5) is known as the Larmor equation, where $\omega_{0}=2\pi v_{0}$ is the angular Larmor resonance frequency, and *γ* is the gyromagnetic ratio, which is a constant for a given nucleus and is proportional to the magnetic strength of the nucleus.

When the nucleus is tipped away from the static magnetic field the orientation of its rotational axis changes leading to precession, as shown in Figure 10(biii). The speed of precession depends on the strength of the static magnetic field and the properties of the nucleus. During the precession motion the nucleus emits RF waves that can be detected by a tuned coil, since the RF waves will induce a voltage in the coils, as shown in Figure 10(biv–bvi). When the RF magnetic field is turned off, the nucleus will continue to spin for a while until it eventually reaches thermal equilibrium and aligns itself again with the direction of the static magnetic field, as shown in Figure 10(bvi). 

**Figure 11:**
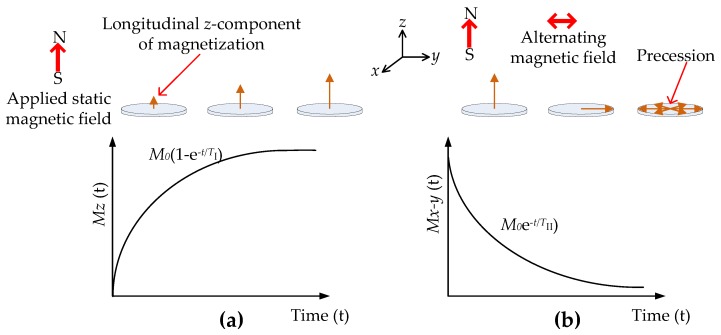
(**a**) Application of a static magnetic field in the *z*-direction results in the spin distributions approach an equilibrium state and the net magnetization of the nucleus being in the *z*-direction. Relaxation time *T*_I_ is known as the longitudinal or spin-lattice relaxation time; (**b**) Application of an alternating magnetic field perpendicular to the static field tips the net magnetization away from the static field onto the *x*-*y* plane leading to precession motion. Relaxation time *T*_II_ is known as the transverse or the spin-spin relaxation time of the nucleus.

**Figure 12:**
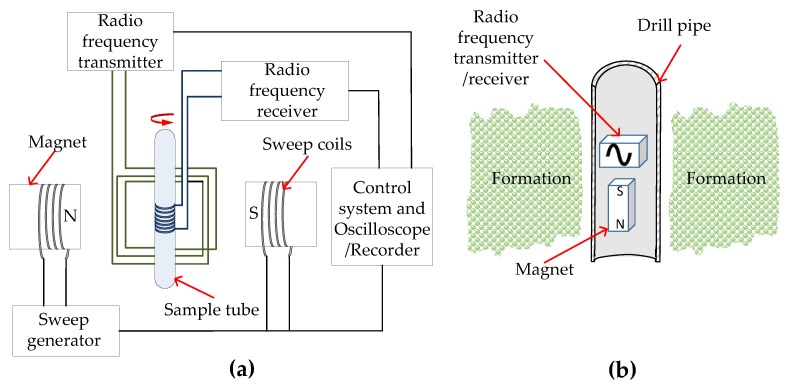
(**a**) Laboratory NMR experimental setup where the sample to be analyzed is placed inside a pair of magnets and transmitter and receiver coils; (**b**) NMR logging setup, referred to as ‘inside out NMR’, where the sample, the formation, to be analyzed is outside the magnets and the transmitter/receiver coils.

During the relaxation process after a static magnetic field is applied, there is a non-radiative transfer of energy where the spin distribution approaches an equilibrium state, as shown in Figure 11a. The time taken to reach equilibrium is called the polarizing time (PT) and *T*_I_ is the relaxation time, also known as the longitudinal or spin-lattice relaxation, where *T*_I_ is the time required for nuclei to reach 63% of the final thermal equilibrium magnetization in the *z*-direction. When an RF pulse is applied the nuclei are tipped away from the static magnetic field, leading to precession motion as shown in Figure 11b. The rate of relaxation is dictated by the time constant *T*_II_. When the RF pulse is turned off the net magnetization in the *x*-*y* plane will decrease exponentially as shown in Figure 12b, and this signal, known as free-induction-decay (FID), is measured by a tuned coil. This FID signal is known as the *T*_II_ relaxation, transverse, or the spin-spin relaxation time. Note that the magnetization is in the *z*, or axial direction, during PT, and in the *x*-*y* plane during the pulse sequence, since the nuclei are tipped away from the static magnetic field by the RF pulses.

### 3.2. NMR for Obtaining Geological Parameters

Evaluating downhole parameters is an important part of the drilling process and nuclear magnetic resonance (NMR) plays a key role in identifying downhole fluid types, formations and oil/gas production potential. One of the main advantages NMR offers for drillers is a non-radioactive option for obtaining the porosity and permeability of rock formations; crucial information to quantify the volume of hydrocarbons present in a formation. Ideally the formations would have oil/gas and large interconnected pores that allow the oil/gas to flow freely out of the formation and up on to the surface.

The earliest studies on NMR properties of fluids in porous media started in the 1950s at a number of oil company research laboratories, and the first patents on NMR well logging was published by Russel Varian and Harold Schwede [55,56]. The first nuclear magnetic logging tool was introduced in 1960 and these early tools measured the FID in the Earth’s magnetic field [57,58,59]. Even though these tools were not commercially successful, the research behind these tools laid a firm foundation for later work. There was a lull in progress of NMR logging tools until Jackson et al. started working on remote ‘inside out’ NMR in the late 1970s and early 1980s, focused on methods of creating regions of homogenous magnetic fields in geological formations external to the NMR device and investigating the sensitivity of NMR detection [60,61,62]. These works triggered further research and development into the ‘inside out’ NMR concept, where, instead of placing the sample inside the NMR apparatus, the apparatus was placed inside the sample, which in this case was the Earth. This required the projection of large static magnetic fields and high frequency RF fields outside the NMR apparatus and into the surrounding geological formations. The first field test with a new design consisting of a permanent magnet and a pulsed RF NMR technique was performed in 1989, and the first commercial tools were available for use the following year [59]. 

Research in the late 1980s and the 1990s showed that complex networks of pore spaces resulted in multi-exponential relaxation, and the distribution of relaxation times were strongly related to the diffusion regimes of the pore spaces [63,64,65,66,67,68,69,70,71]. Algorithms were developed in the late 1990s to implement variable smoothing of distributions so that the smoothing was roughly uniform over the sharp and broad features of quasi-continuous distributions of relaxation times [72]. These research works in multi-exponential relaxation formed the foundation for studies on pore size distributions of different types of porous media and were later adopted to develop algorithms for 2D NMR of diffusion and relaxation to obtain diffusion-diffusion and diffusion-relaxation correlation functions [73,74,75,76]. 

The research efforts in the 1990s and early 2000s also led to pioneering research on compact, mobile devices that could measure NMR signals from objects outside the magnet in a non-invasive manner [77,78,79,80,81,82,83]. These single-sided NMR devices shed light on how to obtain increased field homogeneity, field strength, and even controlled static field gradients. While there were many advantages with these portable single-sided tools there were also problems with significant reduction in field strength and homogeneity, limiting the analysis capability of these tools to only *T*_I_ and *T*_II_ relaxometry. Work was carried out to improve the S/N ratio and optimize the pulse trains by investigating spin dynamics in inhomogenous fields and its effects on diffusion and relaxation [74,84,85]. Based on the results obtained from these works, 2D experiments were performed with a leading diffusion experiment followed by a *T*_II_ sensitive Carr-Purcell-Meiboom-Gill (CPMG) pulse train, where the 2D dataset was processed to obtain 2D maps that correlated diffusion coefficients and transverse relaxation times [73,74,86,87,88]. These works investigating diffusion and relaxation in porous media and determination of pore size and distribution resulted in the ability to unlock the fluid properties from the NMR data and determine the different types of fluid present. 

In order to run wireline NMR tools, first commercially available in the 1990s, drilling had to cease and the drilling assembly had to be pulled out of the well before the wireline NMR could be lowered into it. However, NMR logging while drilling (NMR-LWD) tools, which started appearing in the oil/gas industry in the early 2000s [89,90,91,92,93,94], could determine and identify different fluid phases and their dynamics in geological formations in real-time, while drilling, thus saving time and reducing the cost in a drilling operation. NMR-LWD is rapidly becoming an important logging tool in the oil/gas industry [95] and more recent works on NMR [96,97,98,99,100,101,102,103] continue to drive advancements in NMR-LWD tools to provide more accurate and better characterization of geological formations [104,105,106,107,108,109].

NMR was first used in the petroleum industry as a tool in laboratory core analysis. In such a setup, a formation core extracted from a well is placed inside a sample chamber, as shown in Figure 12a. The sample chamber has two magnets that produce a static magnetic field and a transmitter coil that transmits RF radiation energy via pulses to the sample. The receiver coil surrounding the sample receives the emission of the absorbed RF energy and sends it to an oscilloscope/recorder for analysis. The sweep coils are used to vary the magnetic field over a small range while observing the emitted RF signal from the sample to obtain an NMR spectrum. However, unlike laboratory testing, downhole NMR tools, either run by wireline or LWD, have to measure NMR signals outside the tool, from external geological formations, as show in Figure 12b. In a laboratory setting, the sample is placed inside the magnet and the RF transmitter/receiver coils so that the static magnetic field and RF pulse are projected into the sample. In NMR-LWD, the sample, the formation, is outside the magnet and RF transmitter/receiver coils, so the static magnetic field and the transmitted RF pulse has to be projected out to the sample. NMR well logging is referred to as ‘inside out’ NMR due to the geometry of the magnets and transmitter/receiver coils being inverse to the geometry of a laboratory setting. ‘Inside out’ NMR introduces several complexities such as the need for radially symmetric, high strength static magnetic fields and high frequency RF magnetic fields to ensure sufficient penetration through the well hole and into geological formations, hole size and resultant limits on tool real estate, tool eccentricity inside the hole, drilling fluids, downhole environmental parameters such as, temperature, pressure, pH etc., as along with drilling conditions such as shock and vibration. LWD tools are required to make measurements while rotating, in real or near-real time. Commercially available wireline and LWD tools address these issues and measure NMR signals using different techniques [89,90,91,92,93,95,110,111,112,113,114,115,116,117], but the common objective in all of these tools is to magnetically manipulate and measure NMR signals from the hydrogen nuclei in formation fluids. One important point to note is that the polarization and decay are time-sensitive components and therefore, lateral motion/vibration of the drillstring might result in the magnet and antennas not remaining in one position for long enough to fully polarize a region in the formation and measure the full decay. Such lateral motion/vibration-induced decay primarily affects long *T*_II_ values that may reduce the accuracy of NMR measurements, particularly in light hydrocarbon and carbonate formations. However, magnetometers and accelerometers can be used for quality control of NMR measurements by using the magnetometers and accelerometers to measure lateral motion/vibration-induced parameters such as, frequency, amplitude, trajectory and timing of the vibration, and then utilizing this data to calculate a theoretical maximum *T*_II_ value resolvable during motion and an NMR quality indicator that can be sent with the NMR data [90]. 

**Figure 13:**
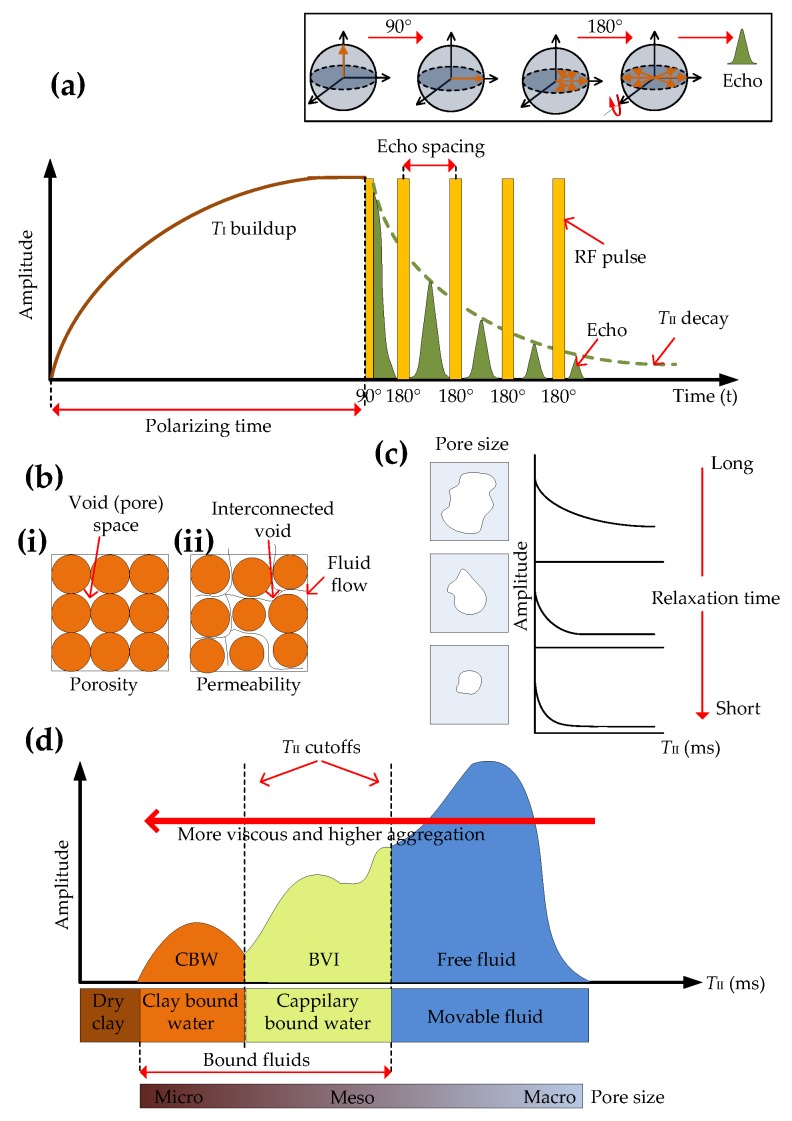
(**a**) Polarization and decay of a nucleus resulting in echo signals (inset shows the spin-echo technique); (**b**) (**i**) Porosity refers to the percentage of void space in a rock formation and (**ii**) permeability refers to the degree of interconnection between these void spaces that allows fluids to flow through the voids; (**c**) In fully water saturated pores larger pore sizes indicate long relaxation times and smaller pores short relaxation times; (**d**) Fluids in formations can be characterized as bound or movable fluids based on *T*_II_ cut-off values. Bound fluids have micro and meso pores and are highly aggregated and more viscous than free fluids, which have larger macro pores. Heavy oils such as tar fall within the bound fluid region whereas intermediate and light oils and gases fall within the free fluid region.

In these tools, the *T*_I_ relaxation time is be measured by either of two methods: either inversion recovery or saturation recovery [54,112,113]. In the inversion recovery method an initial 180° pulse inverts the spins. These spins then gradually relax towards thermal equilibrium. After a given recovery time another 90° pulse is applied to obtain the FID signal. In the saturation recovery method an initial 90° pulse is followed by another 90° pulse after a given recovery time to obtain the FID signal. The *T*_II_ relaxation time can be obtained by the spin echo technique as shown in Figure 13a inset. In the spin-echo technique, a 90° RF pulse tips the nucleus away from the static field onto the transverse *x*-*y* plane and to precession motion. A 180° pulse flips the direction of net spin, which eventually causes the spins with different angular momentums to refocus at a certain time, which is the echo time (the delay between the 90° and 180° pulses). Therefore, the dephasing caused by spin-spin interactions and non-homogeneity of the static magnetic field can be removed and the phase difference between the spins can be maintained, resulting in a strong echo signal. Echo signals are produced between the train of 180° pulses at the same frequency as the RF pulses and decay exponentially with a relaxation time *T*_II_ during the measurement cycle as shown in Figure 13a. 

The amplitude of the echo signals are directly proportional to the net magnetization in the transverse plane, and gives an indication of the quantity of hydrogen nuclei present in a formation. This information is used for porosity measurements, where the porosity, as shown in Figure 13(bi), refers to the percentage of void space found in a rock formation, which could be water, oil or gas, whereas the permeability, as shown in Figure 13(bii), is the degree of interconnection of these void spaces and how easily fluid can flow between the spaces,. The first point of FID or extrapolation of the first echo with accurate exponential fitting is the formation porosity. Assuming a fully water saturated pore, the relaxation time *T*_II_ provides an indication of the pore size, and as Figure 13c shows, large pores have a long *T*_II_ and small pores have a short *T*_II_. Multiple fully water saturated pores have multiple *T*_II_ values for the different pore sizes resulting in a multi-exponential decay curve. While there are many means for nuclei to lose energy and return to equilibrium, one of the main ways they can return to equilibrium, when in a fluid molecule such as a formation fluid for example, is by colliding into the molecule walls. In larger pores these collisions are less frequent than in smaller pores, therefore, nuclei in larger pores have longer relaxation times. Information about the pore size can also be used to estimate the permeability of a formation, since the permeability is proportional to the square of the diameter of the pore. Therefore, the rate of decay of the NMR signal amplitude is used to obtain information about the permeability of the formation. Determination of pore size distribution is another important measurement obtained by NMR since the pore size distribution in a formation can vary significantly leading to a broad distribution. By analyzing this distribution a geological interpretation of the formation can be obtained. 

The relaxation times *T*_I_ and *T*_II_ are influenced by the type of fluid and paramagnetic materials in the formation and diffusion effects of the fluid. For example, when the fluid type is brine, shorter relaxation times indicate smaller pores and longer relaxation times indicate larger pores. The three main mechanisms influencing *T*_II_ relaxation times are grain surface relaxation, relaxation by bulk fluid processes and relaxation from molecular diffusion [113]. Grain surface relaxation is related to pore-size distribution, while bulk fluid and molecular diffusion relaxations are influenced by the type of fluid in the pores. For example, heavy oils and tar have short relaxation times while light oils and gas have longer relaxation times. TII relaxation times can be used to provide estimates of, (i) clay bound water (CBW) and bulk volume irreducible water (BVI), which are called bound fluids, and (ii) moveable or free fluids, in formations, as shown in Figure 13d. The bound fluid region indicates micro and meso pores where the fluid cannot move freely while the movable fluid region indicates larger macro pores. The *T*_II_ cut off times vary with formation lithology. A very heavy oil like tar falls within the CBW spectrum. Heavy oils fall within the CBW and BVI spectrums and intermediate/light oils and gases fall within the free fluid spectrum. Intermediate oils are at the beginning of the free fluid spectrum and gases are towards the end of the free fluid spectrum.

Interpretation of NMR signals using only *T*_II_ relaxation times has its limitations. *T*_II_ relaxation data analyzed by differential spectrum and enhanced diffusion techniques result in 1D analysis of data with the ability to only characterize fluids not quantify them. One of the major drawbacks is the difficulty in distinguishing between water and oil, and, therefore, the inability to quantify the amount of hydrocarbons present. Molecular diffusion opens up the possibility of calculating the water saturation and viscosity of fluids. The diffusion rates of water and gas can be calculated for specific downhole conditions, while the diffusion rate of oil depends on its molecular structure. The resulting 2D cross-plots have data in a 2D space with diffusion and relaxation dimensions [54,113,118,119,120]. This means that heavy oil, conventional oil, light oil, water and gas can be identified and quantified in an oil/gas reservoir. Moreover, the oil/gas water contact measurements using 2D NMR cross-plots are used for field reserve estimates and reservoir field development. It must be noted that *T*_I_ relaxation measurements also provide 1D data sets; additionally 2D *T*_I_–*T*_II_ data sets can be obtained by acquiring a full *T*_II_ echo decay signal during each *T*_I_ acquisition [113]. Moreover, 3D NMR measurements, correlation of fluid properties for *T*_I_, *T*_II_ and D, and 4D NMR measurements, inclusion of the radial distance from the formation wall, are also possible [113]. The accurate profiling of fluids in an oil/gas reservoir has a significant bearing not only on the economic valuation of a reservoir field but also on the surface and production facilities required to optimize the production of a well. 

### 3.3. Field Results

**Figure 14:**
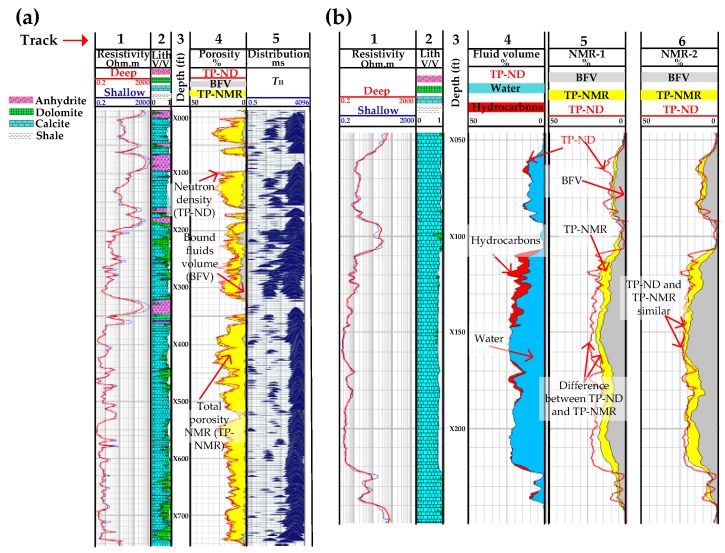
(**a**) Comparison between an NMR and a neutron density tool. Track 1 shows the resistivity of the formation, Track 2 shows the lithology of the formations, such as anhydrite, dolomite, calcite and shale formations and Track 3 shows the depth at which measurements were made. Track 4 shows the porosity measurements, where the red curve shows the total porosity measurements obtained by the neutron porosity tool (TP-ND) and the yellow and gray shaded areas show the total porosity and bound fluid volume, respectively, obtained by the LWD NMR tool (TP-NMR and bound fluid volume (BFV)). Track 5 shows the *T*_II_ distributions of the NMR measurements. The images in Track 4 show that there is excellent agreement between the NMR and neutron-density results when computing total porosity results since the porosity percentages of the red curves and the yellow shaded areas are similar throughout the depths at which the logs were taken; (**b**) An NMR tool was run twice in the hole since measurements obtained during the first run, NMR-1 in Track 5, did not compare well with measurements obtained by the neutron density tool in Track 4. In Track 4 the blue-shaded area corresponds to volume of water and the red-shaded area to hydrocarbons. The red curve seen in Track 5 corresponds to the outline of the total porosity obtained by the neutron density tool (TP-ND) in Track 4 (water+hydrocarbons). The differences between the TP-ND and TP-NMR are shown in Track 5 and were found out to be due to the effect of high salinity drilling fluid used inside the well. After adjusting for high salinity the NMR tool was run inside the well again resulting in a significant improvement in the results as shown in Track 6 [121].

Figure 14a shows porosity estimations from an NMR tool and a neutron-density tool, which is another LWD tool that measures porosity and used for comparison purposes with the NMR tool [121]. Neutron logging tools use a chemical source or an electronic neutron generator to emit neutrons, which collide with hydrogen atoms in a formation and reach thermal equilibrium. The rate at which thermal equilibrium is reached is proportional to the hydrogen concentration, and this information is used to obtain neutron porosity. 

Track 1 in Figure 14a shows the resistivity of the formation. Resistivity measures the degree to which a formation can oppose the flow of electric current. Resistivity is therefore the reciprocal of conductivity, which measures how easily a current can flow in a formation. While fresh water does not conduct electricity, most of the water found in formations have salt ions and therefore conduct electric current and so have low resistivity. Hydrocarbons on the other hand are non-conductive, so the resistivity values increase as the pores in a formation become more saturated with hydrocarbons. The deep and shallow resistivity logs refer to the depths of investigation (DOI), where the shallow logs refer to measurements obtained at the wall of the wellbore, closest to the resistivity logging tool, and the deep logs refer to measurements obtained deep within the formation, furthest away from the logging tool and uncontaminated by the resistivity logging tool. The separation between the shallow and deep logs gives an indication of the diameter of invasion by the drilling fluid and the permeability of the different zones in a formation. It must be noted, however, that resistivity in itself does not always provide an accurate interpretation of a formation and is therefore only used with other logging measurements to aid in the interpretation of formations. Track 2 illustrates the lithology (lith) of the formations, such as anhydrite, dolomite, calcite and shale formations, as a fraction of the rock volume. Track 3 shows the depth at which measurements were made. Track 4 shows the porosity measurements, where the red curve shows the total porosity measurements obtained by the neutron porosity tool (TP-ND), and the yellow and gray shaded areas show the total porosity and bound fluid volume, respectively, obtained by the LWD-NMR tool (TP-NMR and BFV). Track 5 shows the *T*_II_ distributions of the NMR measurements. The images in Track 4 show that there is excellent agreement between the NMR and the neutron-density when computing total porosity results since the porosity percentages of the red curves and the yellow shaded areas are similar throughout the depths at which the logs were taken. 

**Figure 15:**
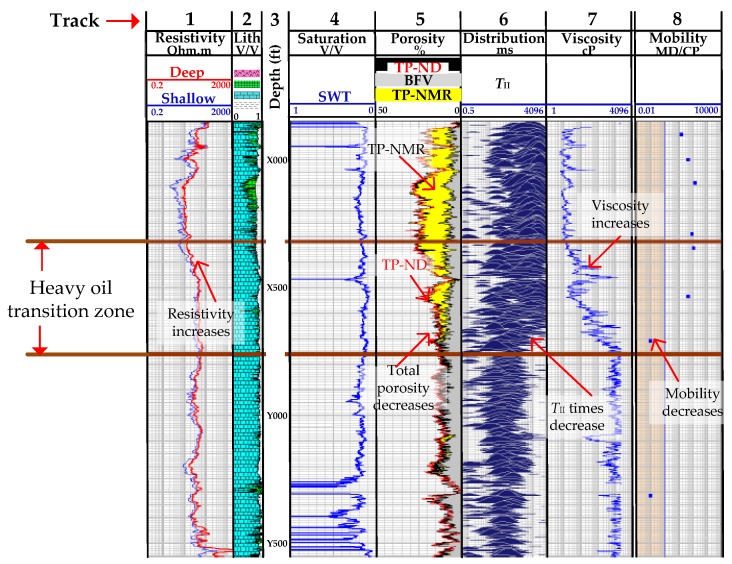
Calculation of viscosity and determination of a heavy oil transition zone. In the heavy oil transition zone shown between the two brown lines, the total porosity measurements obtained by both NMR (TP-NMR) and neutron density (TP-ND) in Track 5 decrease due to pores being saturated with heavy oil, viscosity in Track 7 increases, the mobility in Track 8, which is the ratio of effective permeability to phase viscosity, decreases and the *T*_II_ distribution times become shorter due to restricted molecular motion. A less obvious indicator is resistance in Track 1, which increases slightly in the heavy oil transition zone [121].

Figure 14b shows another comparison between a neutron-density tool and an NMR tool, where Track 1 is the resistivity, 2 the lithology and 3 the depth, as in Figure 14a. Track 4 shows the measurements obtained by the neutron density tool, where the blue-shaded area corresponds to volume of water and the red-shaded area to hydrocarbons. The NMR tool was run twice in the well and Track 5 (NMR-1) shows the measurements obtained during its initial run. The yellow shaded area is the total porosity (TP-NMR) and the grey shaded area is the bound fluid volume (BFV). The red curve seen in this Track corresponds to the outline of the total porosity obtained by the neutron density tool (TP-ND) in Track 4, and is added as a reference to Track 5 for comparison with TP-NMR. However, a clear difference can be seen between TP-ND and TP-NMR, and this difference was found to be due to the effect of high salinity drilling fluid used inside the well. The NMR tool was then pulled out of the well and the tool was adjusted to compensate for high salinity and run inside the well again. Track 6 (NMR-2) shows the measurements obtained during this second run, and it can be seen that the measurements from TP-ND and TP-NMR are in good agreement. Figure 15 shows the estimation of viscosity and how this information is used to identify a heavy oil transition zone in the reservoir. In the heavy oil transition zone shown between the two brown lines the total porosity measurements obtained by both NMR (TP-NMR) and neutron density (TP-ND) in Track 5 decrease due to pores being saturated with heavy oil, viscosity in Track 7 increases, the mobility in Track 8, which is the ratio of effective permeability to phase viscosity, decreases and the *T*_II_ distribution times become shorter due to restricted molecular motion. A less obvious indicator is resistance in Track 1, which increases slightly in the heavy oil transition zone. 

**Figure 16:**
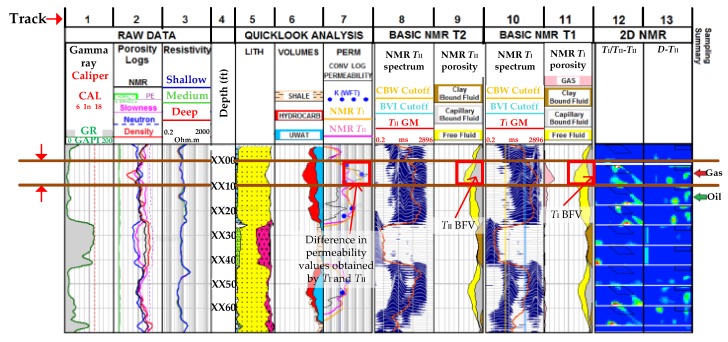
Basic and 2D NMR analyses of a well. *T*_I_ and *T*_II_ spectrum data (Tracks 8 and 10) and 1D partial porosity data (Tracks 9 and 11) obtained by inversion techniques is referred to as basic data, where each element of this spectrum is a volume of fluids with a given *T*_I_ or *T*_II_ relaxation time. 2D NMR data was obtained by the inversion of multi-echo train data sets, and as shown by the 2D images in Tracks 12 and 13, this well contains gas and high gas/oil ratio oil. As shown in the boxes outlined in red in Tracks 7, 9 and 11, at depths XX00–XX10, between the two brown lines, there is a noticeable difference between the *T*_I_ and *T*_II_ bound fluid volumes and their corresponding permeability estimates. While this is due to the gas signal being pushed below the BVI cutoff line due to the effect of diffusion, *T*_I_ on the other hand is not affected by diffusion and therefore, provides the correct bound fluid estimation. The 2D NMR plots estimate the volume of gas present by summing the NMR signals within a ’box’ centered on the theoretical position as shown in Tracks 12 and 13. The gas volume at XX10 was predicted to be a gas-oil contact and this was later confirmed by further tests [118].

Figure 16 shows basic and advanced 2D NMR analyses on data obtained by an NMR tool [118]. Track 1 shows gamma ray and caliper measurements. Gamma ray measurements provide information about the formation lithology (Track 5) in gamma-ray, American Petroleum Industry (GAPI) units while caliper measurements provide information on the width of the well in inches. Tracks 4, 5 and 6 provide depth, lithology and fluid volume information. *T*_I_ and *T*_II_ spectrum data (Tracks 8 and 10) and 1D partial porosity data (Tracks 9 and 11) obtained by inversion techniques is referred to as basic data, where each element of this spectrum is a volume of fluids with a given *T*_I_ or *T*_II_ relaxation time. CBW, BVI and free fluids are calculated by the summation of partial porosities within different ranges of the CBW, and BVI cut off values in the spectrum (Tracks 8–11). Track 7 shows the permeability, estimated by NMR porosity analysis. 2D NMR data was obtained by the inversion of multi-echo train data sets, and, as illustrated by the 2D images in Tracks 12 and 13, this well contains gas and high gas/oil ratio oil. 

As shown in the boxes outlined in red in Tracks 7, 9 and 11, at depths XX00–XX10, between the two brown lines, there is a noticeable difference between the *T*_I_ and *T*_II_ bound fluid volumes and their corresponding permeability estimates. While this is due to the gas signal being pushed below the BVI cutoff line due to the effect of diffusion, *T*_I_ on the other hand is not affected by diffusion and therefore, provides the correct bound fluid estimation.The 2D NMR plots estimate the volume of gas present by summing the NMR signals within a ‘box’ centered on the theoretical position, as shown in Tracks 12 and 13. The gas volume at XX10 was predicted to be a gas-oil contact, a fact later confirmed by further tests.

**Figure 17:**
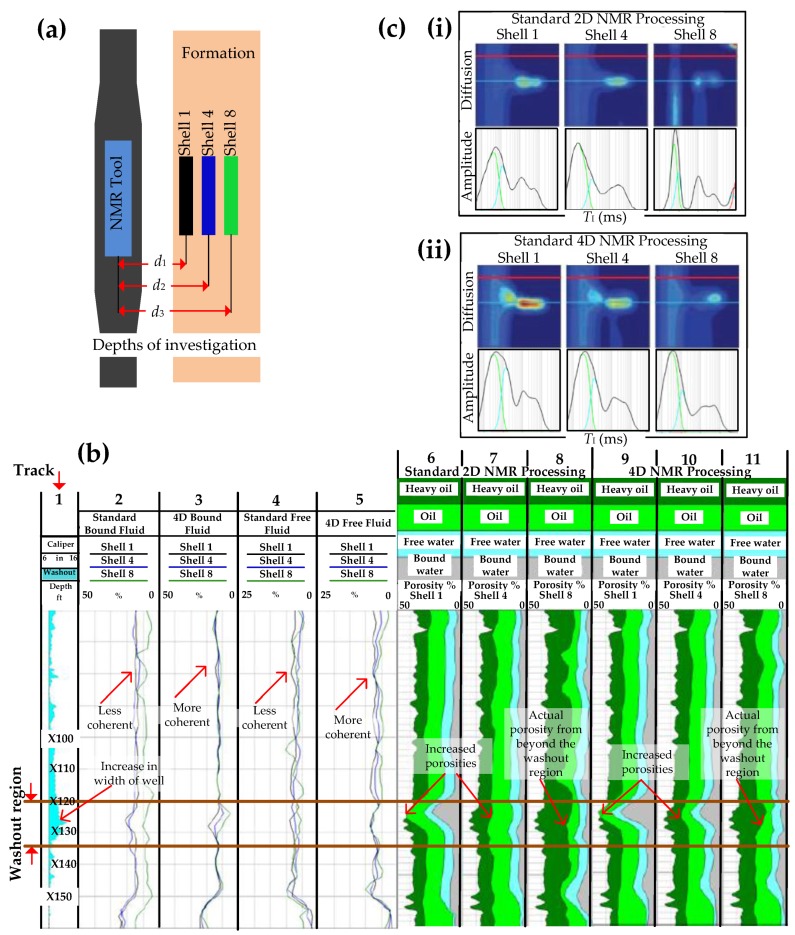
4D NMR processing. (**a**) 4D measurements incorporate a fourth dimension, the radial distance *d*, from the tool to the formation leading to acquisition of data at multiple depths of investigation (*d*_1_, *d*_2_ and *d*_3_); (**b**) Bond and free fluid porosity measurements are shown from Tracks 2–5. It is difficult to distinguish between the standard bound fluid volumes measured by the 3 shells (shells 1, 4 and 8) in Track 2 and as well as for free fluid volumes measured by the three shells in Track 4. However, constraining the bound volumes and reassigning the porosity contributions resulted in a much clearer picture with less deviation between the results obtained by the 3 shells, as shown in Tracks 3 and 5. Fluid volumetric analysis results are shown from Tracks 6–11. Fluid properties were affected by hole a washout from X120 to X135 ft (between the two brown lines), which resulted in a larger diameter of the well, shown by the caliper readings in Track 1 and the increased porosities from shallower shells (Tracks 6, 7, 9 and 10). Shell 8 in Tracks 8 and 11 were from beyond the washout and provided more accurate measurements; (**c**) *D*-*T*_I_ maps; (**i**) Standard 2D NMR processing shows shells 1 and 4 having similar bound fluid volumes but shell 8 having a lower bound fluid volume than 1 and 4, even though the bound fluid volumes are expected to be the same across all shells; (**ii**) 4D NMR processing provides a more accurate measurement for shell 8 by constraining the fluid volume to be the same below a *T*_I_ cutoff value and reapportioning the porosity to account for the bound fluid volume [113,122].

Figure 17 shows an example of how 4D NMR processing results in more accurate estimation of fluid volumes and properties [113,122]. While 3D NMR measurements obtain echo data at multiple polarization times (for *T*_I_) and multiple echo spacings (for diffusion) to construct 3D maps, 4D measurements incorporate a fourth dimension, the radial distance *d* from the tool to the formation, which results in the acquisition of data at multiple depths of investigation, as shown in Figure 17a. The shells refer to the depth of investigation (DOI) inside the formation, where shell 1 is closest to the tool, at a distance of *d*_1_, followed by shell 4, at a distance of *d*_2_, and shell 8, which is the furthest away at a distance of *d*_3_. Data from the closest shell to the formation was used to correct data from the shells deeper inside the formation, thereby compensating for the decreasing magnetic fields and echo signals resulting in poorer signal-to-noise ratios in deeper shells. A major assumption made in 4D NMR processing is that the bound fluid volume does not change with the DOI since the invasion of filtrate from drilling fluids does not generally change clay and capillary-bound fluids. Constraining the bound fluids in the deeper shells to be equal to the bound fluid in the more accurate shallower shells, and reassigning the total porosity across the fluid spectrum, leads to more accurate NMR analysis. 

Figure 17b shows the bound and free fluid porosity measurements from Tracks 2 to 5, where Track 1 includes caliper measurements and the depth, 2 and 4 are the standard bound and free fluids, respectively, and Tracks 3 and 5 are the 4D bound and free fluids, respectively. It is difficult to distinguish between the bound fluid volumes measured by the 3 shells (shells 1, 4 and 8) in Track 2 as well as the free fluid volumes measured by the three shells in Track 4. However, constraining the bound volumes and reassigning the porosity contributions results in a much clearer picture where there is less deviation between the results obtained by the 3 shells, as shown in Tracks 3 and 5. Tracks 6–11 in Figure 17b shows the fluid volumetric analysis results and shows an example where fluid properties were affected by hole conditions from X120 to X135 ft (between the two brown lines). In this case a hole washout results in a larger diameter of the well, as shown by the caliper readings in Track 1, and the increased porosity measurements from shallower shells (Tracks 6, 7, 9 and 10). Shell 8 measurements in Tracks 8 and 11 are from beyond the washout and provide more accurate data. Figure 17c illustrates how the diffusion and *T*_I_ maps used for saturation computation for each shell demonstrate the effectiveness of 4D processing. Standard 2D NMR processing in Figure 17(ci) shows shells 1 and 4 having similar bound fluid volumes but shell 8 having a lower bound fluid volume than 1 and 4, even though the bound fluid volumes are expected to be the same across all shells. 4D NMR processing in Figure 17(cii) provides a more accurate measurement for shell 8 by constraining the fluid volume to be the same below a *T*_I_ cutoff value and reapportioning the porosity to account for the bound fluid volume.

## 4. Discussion: Research Opportunities for Other Types of Magnetic Sensors

The harsh and hostile downhole environment is characterized by high temperature, high pressure, acidic and corrosive environments, as well as high torque, shock and vibration effects resulting from the drill bit grinding through formations. Based on the typical parameters encountered by drilling tools downhole shown in Table 2, it is safe to state that not many applications expose sensors and instrumentation to such severe conditions. The failure of sensors could result in drilling companies incurring a significant loss since, in such a situation the drilling has to be stopped, the whole drilling assembly has to be pulled out of the well for repairs and maintenance and then has to be run inside the well again. FGMs and NMR sensors are designed, fabricated and packaged to survive these conditions. However, FGMs are bulky, since they involve ferromagnetic cores and many windings, which take up a significant amount of space in a drillstring, where space is at a premium [2,123]. The ferromagnetic core design, such as the geometry, materials, and the intrinsic losses in the core, along with the errors pertaining to the windings of the coils, also influence the performance, and FGMs are not easy to calibrate [2,124,125]. Another key limitation is the high power consumption of FGMs; up to 1 W. Power usage in a downhole environment has to be managed carefully due to the inaccessibility from the surface. The power to downhole sensors and instrumentation is generally provided by a turbine generator and/or battery pack. The turbine is used to generate electricity by the flow of drilling fluids in the drillstring assembly, and batteries provide continuous, finite power that is rechargeable by the turbine. However, power usage of sensors and instrumentation such as FGMs dictate the turbine/battery pack design to be expensive and occupy a significant amount of space in a drillstring. The advent of microelectromechanical systems (MEMS) technology has allowed the scaling down of mm size devices into the micro-nano range. This provides the opportunity to package and fit these sensors into smaller areas in a drillstring as well as reduce the effects of shock and vibration. The smaller sizes allow sensor arrays thus, increasing the resolution of measurements, and also seamless integration with other electronic components leading to ‘system on chip devices’ that can be mass produced. MEMS devices have low power requirements and the small size of the sensors makes it more tolerant to mechanical shocks and vibrations. Recent research has also shown that MEMS sensors can match much larger and higher power FGMs when it comes to noise performance [126]. FGMs can be scaled down using microfabrication techniques but several challenges remain as explained in Section 2.1. This gives rise to the question; what other types of magnetic sensors have the potential to be used in downhole environments?

Several magnetic sensors have the potential to be used in downhole environments and magnetoresistive (MR) sensors [127,128,129,130,131] can be considered the frontrunners in this regard due to their high sensitivity, high temperature tolerance, temperature stability, low power consumption and well established method of fabrication on silicon substrates. Magnetoresistance refers to the change in electrical resistance of a material or system of materials in response to an applied magnetic flux density. MR sensors can measure the Earth’s magnetic field but can also accurately measure magnetic fields from permanent magnets and soft ferromagnetic structures and magnetic fields generated by electric currents. This gives rise to the use of these sensors not only as directional sensors but as potential sensors for measurement of rotation speed, angle and positioning of the drilling assembly and drill bit. For example, by utilizing a ring of permanent magnets as a reference and a MR sensor/sensor array it may be possible to measure the drill bit/drilling assembly speed, torque, angle and position. These parameters can be optimized to increase the rate of penetration of the drillbit into the formations. Moreover, these measurements can be used generating drilling road maps for different formations in different oil/gas fields therefore, optimizing drilling efficiency. MR sensors have been used in commercial applications operating at temperatures up to 225 °C, pressures up to 20,000 psi and mechanical shocks up to 1500 g [132]. Recent research has also shown that MR sensors can be used to calculate the length of directional wells, where the length can be used in fitting the drilling profile, computing the deviation, designing the drilling profile, and developing an algorithm for automated directional drilling [133], to measure NMR signals at room temperature [134] and operate at temperatures up to 175 °C for 5000 h with low energy consumption [128]. Apart from meeting the criteria for use in harsh environments low power consumption is a key advantage in these sensors since power is at a premium in downhole environments. This feature can be exploited to design and fabricate autonomous, wireless sensors, which are important in poorly accessible areas like the downhole environment, since the sensors and electronics are energy efficient and most of the power will then only be used for wireless transmission. 

Hall effect sensors create a Hall voltage across a p-type semiconductor when the continuous electrons running through it will move to one side of the sensor when it is exposed to an external magnetic field [1,2,135,136]. Hall effect sensors can also be prospective downhole magnetic field sensors but they are less sensitive than magnetoresistive sensors, are affected by the piezo effect leading to a lower offset stability than the magnetoresistive sensors and are less mechanically robust than MR sensors. Also, the performance of Hall sensors decrease rapidly at temperatures above 150 °C and currently there are no commercially available Hall sensors that can operate above 200 °C. However, Hall sensors have a higher linear range compared with magnetoresistive sensors, can be integrated onto a single chip with electronics and unlike MR sensors, which can measure a maximum of 180° around a circle due to its periodic behaviour, Hall sensors can measure a full 360° around a circle. Recent research works show the progress made in developing Hall effect sensors for extreme environments [137,138,139,140] but Hall effect sensors need further research to build up on these preliminary investigations with the aim of commercialization and to increase its stability under severe mechanical effects.

Giant magnetoimpedance (GMI) sensors are another type of magnetic sensor that can be exploited for use in downhole environments. GMI refers to the change in the complex impedance of a ferromagnetic conductor with a high frequency current flowing through it when exposed to an external magnetic field. GMI structures are generally in the form of a microwire, thin film or thin ribbon [1,141,142]. Compared with MR and Hall sensors GMI is the least established magnetic sensor and there are only a few commercial GMI sensors currently available in the market and these sensors only operate at temperatures below 100 °C. One of the major drawbacks of GMI sensors is the high frequency they work at, especially the drive current that is generally in the GHz range making it difficult to integrate it with signal processing electronics into a single chip. Moreover, they have higher temperature offset drifts compared to MR and Hall sensors. However, GMI sensors are robust devices that have high sensitivity and can be made into flexible and passive wireless sensors [143,144,145] making them an attractive area of research for downhole environments. Even though the sensitivity of GMI sensors decrease with temperatures they still remain high enough at downhole temperatures [146,147] and if the temperature offset can be sufficiently compensated they can be potentially be used in downhole applications. Current commercial applications include electronic compasses in smart phones and in biomedical applications [148].

## 5. Conclusions

The principles and applications of two types of magnetic sensors, fluxgate magnetometers (FGMs) and nuclear magnetic resonance (NMR) sensors, used in the oil/gas industry have been described in this paper. The method of utilizing FGMs in a drilling assembly to accurately obtain well trajectory information while drilling, to navigate a well to a desired zone has also been described. Additionally, the technique of using NMR sensors to obtain information regarding downhole geological properties while drilling, which enables the characterization and quantification of downhole fluids in reservoirs, has been presented. Finally, a discussion on magnetic sensors that present research opportunities for utilization in downhole environments has been provided. 

## Figures and Tables

**Table 1:** Typical parameters of fluxgate magnetometers.

Field Range	Sensitivity	Linearity	Temperature Coefficient	Size	Noise	Frequency
10 pT–2 mT	20–50 mV/μT	<10 ppm	0.25 nT/°C	mm	15 pT/√Hz	10 kHz

**Table 2:** Typical downhole parameters.

Temperature	Pressure	pH	Vibration	Shock
125–230 °C	15,000–30,000 psi	2–5	30 g peak at 50–1000 Hz	1000 g, 0.5 ms, Half sine
